# Practical Handbook of the Pathology of the Skin

**Published:** 1904-12

**Authors:** 


**Practical Handbook of the Pathology of the Skin.** By J. M. H. Macleod, M.A., M.D. Pp. xxiv., 408. London: H. K. Lewis. 1903.

This work claims to be an introduction to the histology, pathology and bacteriology of the skin, with special reference to technique. It is primarily written for the use of students, but it is a work which no one interested in dermatology can well leave unstudied, for it is the only book of its kind yet written in this or any other language. It supplies a want which has been long felt, in that it is a reliable text-book in which the work of the best-known authorities has been judiciously compiled and classified, and yet at the same time one feels throughout that the facts stated have been proved, and that a master-hand has held to that which is good.

The future of dermatology lies very largely in a correct appreciation of the pathological conditions present in the various dermatoses. As Dr. Arthur Whitfield truly said at the Oxford meetings: "At the present day those who devote their attention to the special study of skin diseases have been directing their attention to the macroscopic and microscopic morbid anatomy of these, and by careful study of the micro-organisms present have done much to elucidate many problems of etiology."

At the same time one is struck very forcibly by the breadth of field opened up in this view of the subject and by the small amount of ascertained detail in even the commonest of affections. The future will add largely to our knowledge in this direction, and it will certainly help largely to this end that the present-day student can start with the basis so clearly and ably set forth in this book.

Among the references there is a singular dearth of English authorities, and this alone speaks for the need of advance amongst English dermatologists along the lines laid down. The book seems to herald a new era in what may indeed prove useful in general as well as special pathology. The skin is easily examined, and its changes readily appreciated.

The book will find its place in the study as well as the laboratory, and it is difficult to praise too highly one so well compiled, so interestingly written, and so delightfully placed before the reader.

No pains have been spared to reproduce the drawings, coloured and otherwise, which alone speak for the patient and careful skill of the author.

There are chapters on subjects of a more general character, but which, however, bear strongly upon the skin, and add to the usefulness of the work. It is to be hoped that the subsequent editions will be kept up to date and produced in as praiseworthy a manner as the original.

